# Systemic humoral immune responses and metabolic parameters in chronically *Neospora caninum*-infected primiparous Holstein heifers during the transition period

**DOI:** 10.3389/fimmu.2026.1898624

**Published:** 2026-09-03

**Authors:** Piedad C. Rivas-Lopez, Evangelina Miqueo, María E. Primo, María F. Farcey, Karen D. Moran, Beatriz Valentini, Franco Fiorani, Claudia G. Morsella, Karina M. Cirone, Lucía M. Campero, Dadin P. Moore, María G. Bilbao, Jenny J. Chaparro-Gutiérrez

**Affiliations:** 1 Faculty of Agricultural Sciences, National University of Mar Del Plata, Balcarce, Argentina; 2 Laboratory of Veterinary Immunology and Parasitology, National Institute of Agricultural Technology (INTA), Rafaela Agricultural Experimental Station, Santa Fe, Argentina; 3 National Research Council (CONICET), General Pico, Argentina; 4 National University of La Pampa, Faculty of Veterinary Sciences, General Pico, Argentina; 5 Institute of Innovation for Agricultural Production and Sustainable Development (IPADS), Balcarce, Argentina; 6 CCT Patagonia Confluencia (Confluencia, Argentina), Confluencia, Argentina; 7 Center for Basic and Applied Research in Veterinary Medicine (CIBAV Group), School of Veterinary Medicine, Faculty of Agricultural Sciences, University of Antioquia (UdeA), Medellín, Colombia

**Keywords:** *Neospora caninum*, transition period, IgG, avidity, dairy cattle

## Abstract

Bovine neosporosis has emerged as a major cause of reproductive losses in dairy cattle worldwide, representing a relevant concern for animal welfare and health. The transition period in dairy cattle is characterized by physiological, metabolic and immunological changes. However, the specific humoral immune response against *Neospora caninum* has been only partially characterized during this critical period, raising the hypothesis that transition-associated physiological changes may influence the humoral response to chronic *N. caninum* infection. The aim of this study was to characterize changes in *N. caninum*-specific total IgG, IgG_1_/IgG_2_ ratio, and avidity in serum samples from chronically infected Holstein heifers (n = 7) at prepartum, calving, and postpartum sampling times. Additionally, temporal profiles of NEFA, cholesterol, GOT, GPT; total proteins and albumin between chronically *N. caninum*-infected and seronegative Holstein heifers (n = 5) were described. Specific total IgG antibody levels declined progressively from prepartum to postpartum (p < 0.05), with intermediate values observed at calving. The IgG_1_/IgG_2_ ratio was significantly reduced at calving compared to prepartum (p < 0.05), indicating a transient shift in the IgG subclass balance around calving. No significant differences were detected in avidity. Regarding metabolic indicators during the transition period, no significant interactions between serostatus and time were observed for any parameters except for NEFA concentrations (p < 0.05). The present study demonstrates that chronically *N. caninum*-infected Holstein heifers undergo measurable changes in humoral immune parameters across the transition period. Total specific IgG declined progressively from prepartum to postpartum, and the IgG_1_/IgG_2_ ratio was transiently reduced at calving, indicating a shift in antibody subclass balance rather than a uniform decline across all IgG-related parameters. Antibody avidity remained stable throughout the sampling period. Noteworthy, this humoral response to *N. caninum* infection did not appear to modify the metabolic status characteristic of the transition period: significant serostatus × time interaction was observed for cholesterol, GOT, GPT, total proteins, or albumin. This indicates that the immunological changes associated with chronic *N. caninum* infection occur largely independently of the metabolic adaptations that dairy heifers undergo during the periparturient period, and do not appear to exacerbate the metabolic stress typically observed during this stage.

## 1 Introduction

*Neospora caninum* is an obligate intracellular apicomplexan protozoan and a major cause of reproductive failure in cattle worldwide, recognized as the most diagnosed cause of abortion in areas with an intensive dairy industry (1, 2). The parasite can be transmitted both horizontally, through ingestion of oocysts shed by definitive canid hosts, and vertically via transplacental transmission from chronically infected dams to their offspring (3, 4). Both innate and humoral immune parameters in colostrum and calves from heifers experimentally infected with *N. caninum* were described recently (5). At delivery, serum and colostrum samples from infected group evidenced specific IgG anti-*N. caninum*. Infected heifers showed IgG_1_/IgG_2_ ratios <1 and high avidity specific IgG. As expected, colostrum samples of these animals exhibited a high IgG_1_ concentration and elevated avidity values. Three out of 4 calves from *N. caninum*-infected heifers had specific IgG with IgG_1_/IgG_2_ ratios >1 and lower avidity values before colostrum intake. Interestingly, both IgG_1_/IgG_2_ ratios and avidity values increased in seropositive calves after colostrum intake. Whether these changes in the antibody mediated immune response occur in naturally infected cattle remain to be determined.

The transition period in dairy cattle, defined as the interval extending from three weeks before to three weeks after calving, represents one of the most physiologically demanding stages in the productive life of heifers and cows (6). During this period, the increased demand for energy and nutrients for colostrum and milk synthesis, together with a reduction of feed intake, resulted in negative energy balance, stimulating the mobilization of body fat in the form of non-esterified fatty acids (NEFA) (7). Beyond these metabolic disruptions, inflammation at calving and changes in immunocompetence, redox balance, and mineral metabolism are hallmarks of this critical window. Several components of the host defense system are altered during this period while increased glucocorticoids secretion contributes to immunosuppression with high incidence of health disorders like mastitis, fatty liver disease, ketosis, retained placenta, and metritis (8).

Despite this progress, the humoral immune response during the transition period in chronically *N. caninum*-infected heifers has not been fully characterized. Given the profound immunological changes, occurring during this period, both the concentration and functional quality of anti-*N. caninum* antibodies are expected to change around calving. Furthermore, parameters related to nitrogen metabolism (total protein, albumin), adipose tissue metabolism (cholesterol, NEFA) and hepatic transaminases (GOT, GPT) have been proposed as key indicators of liver function during the transition period, however their temporal profiles have not been compared between *N*. *caninum* chronically infected and seronegative heifers (9; Kessler et al., 20 14). The aim of this study was to characterize changes in *N. caninum*-specific total IgG, IgG_1_/IgG_2_ ratio, and avidity in serum samples from chronically infected Holstein heifers (n = 7) at prepartum, calving, and postpartum sampling times. Additionally, temporal profiles of NEFA, cholesterol, GOT, GPT; total proteins and albumin between chronically *N. caninum*-infected and seronegative Holstein heifers (n = 5) were described.

## 2 Materials and methods

### 2.1 Animals and experimental design

The Holstein heifers used in this study belong to a dairy herd of 200 animals located INTA-Balcarce, Argentina. The herd was maintained on pasture with daily supplements of corn and silage. The herd was brucellosis and tuberculosis free and a vaccination program against foot and mouth disease was routinely performed. The mean calving to conception and calving to calving intervals were 65 and 370 days, respectively. Seroepidemiologic data and pathological studies performed on the herd have revealed that neosporosis has been endemic for 25 years, with a prevalence of 22%, vertical transmission rate of 65% and a horizontal transmission rate of <3% and sporadic abortions caused by *N. caninum* (2). Before the start of the postpartum, the animals were screened to detect those that were seropositive. Blood samples were taken twice a year prior to breeding, and their serostatus was determined by a commercial competitive ELISA (c-ELISA, ID Screen^®^ *Neospora caninum* Competition), following the manufacturer’s instructions. Heifers were had adequate genital development and were cycling at the start of the trial. Routine clinical examination, pelvic area measurements and vaccination with inactivated vaccines against Bovine Viral Diarrhea Virus-1 (BVDV-1) and Bovine Herpesvirus-1 (BHV-1) (Biogenesis ™, Argentina) were performed twice, two months before breeding. Heifers were oestrus synchronized with cloprostenol according to the manufacturer’s instructions (Estrumate, Coopers™, Germany). Animals were allocated to pens for natural mating using 3 dairy bulls for 7 days, with water and food supplied ad libitum. Forty-five days after breeding, 7 seropositive and 5 seronegative Holstein heifers were selected for the trial. For the present analysis, three sampling time points were defined relative to parturition: prepartum (approximately 4 weeks before the expected delivery date), calving (within 24 hours of calving), and postpartum (approximately 4 weeks after calving). Serum samples from all animals were obtained for serological analysis and for *N. caninum* DNA detection in whole blood samples.

### 2.2 *Neospora caninum* evaluation in blood samples

Blood samples were collected with EDTA (Wiener Laboratorios S.A.I.C., Rosario, Argentina) by jugular vein puncture were obtained at three sampling time points (see section 2.1). DNA was extracted with a commercial kit (Wizard^®^ Genomic DNA Purification Kit) following manufacturer instructions, and a nested PCR (nPCR) targeting the ITS-1 region *of N. caninum* was performed to evaluate the parasite presence (10). DNA from cultured tachyzoites (NC-1 strain) was used as a positive PCR control and negative controls (PCR grade water) were included in each PCR reactions.

All procedures were approved by the Animal Ethics Committee at INTA Balcarce (CICUAE 687082/2022).

### 2.3 Systemic humoral immune responses: specific total IgG, IgG_1_/IgG_2_ ratio and avidity index value

#### 2.3.1 Specific total IgG

Serum samples were analyzed using a commercial competitive ELISA (c-ELISA, ID Screen^®^ *Neospora caninum* Competition), following the manufacturer’s instructions with modifications. Briefly, the final antibody titer was determined by serial two-fold dilutions of each serum sample (1:2, 1:4, 1:8, 1:16, 1:32, 1:64, and so on). The optical density (OD) was recorded for each dilution, and the endpoint titer was defined as the highest serum dilution at which the OD value still differed from that of the subsequent (higher) dilution. Once a further two-fold dilution no longer produced a change in OD relative to the preceding dilution, the reaction was considered to have reached a plateau, and the last dilution preceding this plateau was recorded as the final titer. For example, if the OD obtained at a 1:64 dilution did not differ from that obtained at 1:32, the final specific IgG titer against *N. caninum* was reported as 1:32. Optical density values were measured with a microplate reader (Bioteck, US).

#### 2.3.2 Specific IgG sub-isotypes

Specific IgG_1_ and IgG_2_ were assessed by indirect in-house ELISA using anti-bovine IgG_1_ or IgG_2_ monoclonal antibodies (MA5–16736 or MA5-16737, Invitrogen, Waltham, MA, USA) both diluted 1:50, and recombinant tSAG1 as the coating antigen (11). Each serum sample, diluted 1:10, was simultaneously evaluated with both monoclonal antibodies on the same plate. Results were expressed as the IgG_1_/IgG_2_ OD ratio (12).

#### 2.3.3 Specific IgG avidity

A commercial indirect iELISA (ID Screen^®^ Neospora *caninum* Indirect, IDVet, France) was used with slight modifications for avidity determination. Briefly, each serum sample was analyzed in 2-fold titration rows (5 dilutions). Each serum sample was processed in duplicate, where only odd-numbered rows were treated with urea. For urea treatment, 250 µL of 6 M urea in phosphate-buffered saline containing 0.05% tween 80 was applied to remove low-avidity antibodies from their binding sites. For each serum, 2 endpoint titers (1 after incubation with urea and 1 without urea incubation) were calculated using A450 and the cut-off was independently established for each sample at the OD where the dilution curve reaches linearity. The avidity index value, expressed as percentage of avidity, was calculated as: (endpoint titer with urea/endpoint titer without urea) × 100 (13).

### 2.4 Metabolites

Serum metabolite concentrations were determined by spectrophotometry using an Intelligent Clinical Chemistry Analyzer (InCCA, Diconex SA, Quilmes, Argentina). Commercial test kits, standards, and quality controls for cholesterol, GOT, GPT, total protein, and albumin were obtained from Wiener Laboratorios (Rosario, Santa Fe, Argentina) and used according to the manufacturer’s instructions. Briefly, each metabolite assay was calibrated utilizing Calibrator A plus, and quality control assessments were performed using the two-level Standatrol S-E (Wiener Lab). NEFA concentrations were quantified using a commercial kit (Randox Lab Ltd, UK). All samples were analyzed within the same batch on the same day.

### 2.5 Statistical analysis

All statistical analyses were performed in R (R Core Team, version 4.5.2). Data were first visually inspected and checked for normality and homoscedasticity using residual diagnostics.

Humoral immune responses, including reciprocal IgG titre (IgG), IgG_1_/IgG_2_ ratio, and avidity index, were analyzed in seropositive animals using linear mixed-effects models fitted with the lme4 package. For each response variable, sampling time (prepartum, partum, and postpartum) was included as a fixed effect, and animal identity was included as a random intercept to account for repeated measurements. Significance of fixed effects was assessed using Type II Wald chi-square tests. Model assumptions were evaluated through residual diagnostics, confirming homoscedasticity and normality in all cases. Estimated marginal means (least-squares means) were obtained using the emmeans package. When a significant effect of time was detected, pairwise comparisons among time points were performed with Tukey adjustment for multiple comparisons. Differences among means were further summarized using compact letter displays (CLD; a, b, ab), where groups sharing at least one letter were not statistically different (p < 0.05). For antibody titre and avidity index, estimates were back-transformed from the log scale for presentation.

Variables showing non-normal distribution (NEFA, GOT, and GPT) were log-transformed prior to analysis. To evaluate the association between metabolic profile and serostatus, linear mixed-effects models were fitted using the lme4 package. Each metabolite (NEFA, GOT, GPT, albumin, total protein, and cholesterol) was analyzed as a dependent variable, including serostatus (positive vs. negative), sampling time (prepartum, calving, and postpartum), and their interaction as fixed effects. Cow was included as a random intercept to account for repeated measures.

Model selection was performed by comparing full models (including the interaction term) with reduced models (additive effects only) using likelihood ratio tests. When the interaction was not significant (p > 0.05), the reduced model was retained.

To evaluate the robustness of the interaction between serostatus and sampling time on NEFA concentrations, a leave-one-out sensitivity analysis was performed. Each animal was sequentially excluded from the dataset, and a linear mixed-effects model was refitted to the reduced data using the lme4 package in R. The model included NEFA as the response variable, serostatus, time, and their interaction as fixed effects, and animal identity as a random intercept.

For each iteration, the significance of the serostatus × time interaction was extracted using Type II Wald chi-square tests. This procedure generated a distribution of p-values reflecting the influence of individual animals on the inference of the interaction effect. The interaction remained consistent across all iterations, indicating that results were not driven by any single subject.

Estimated marginal means were obtained using the emmeans package. Pairwise comparisons between serostatus groups within each sampling time were conducted, and significance was declared at p < 0.05. Data are presented as least-squares means ± standard error (SE).

## 3 Results

### 3.1 Animals and calves

All heifers delivered healthy calves. No placental retention was recorded.

### 3.2 Humoral immune responses

**Figure 1:**
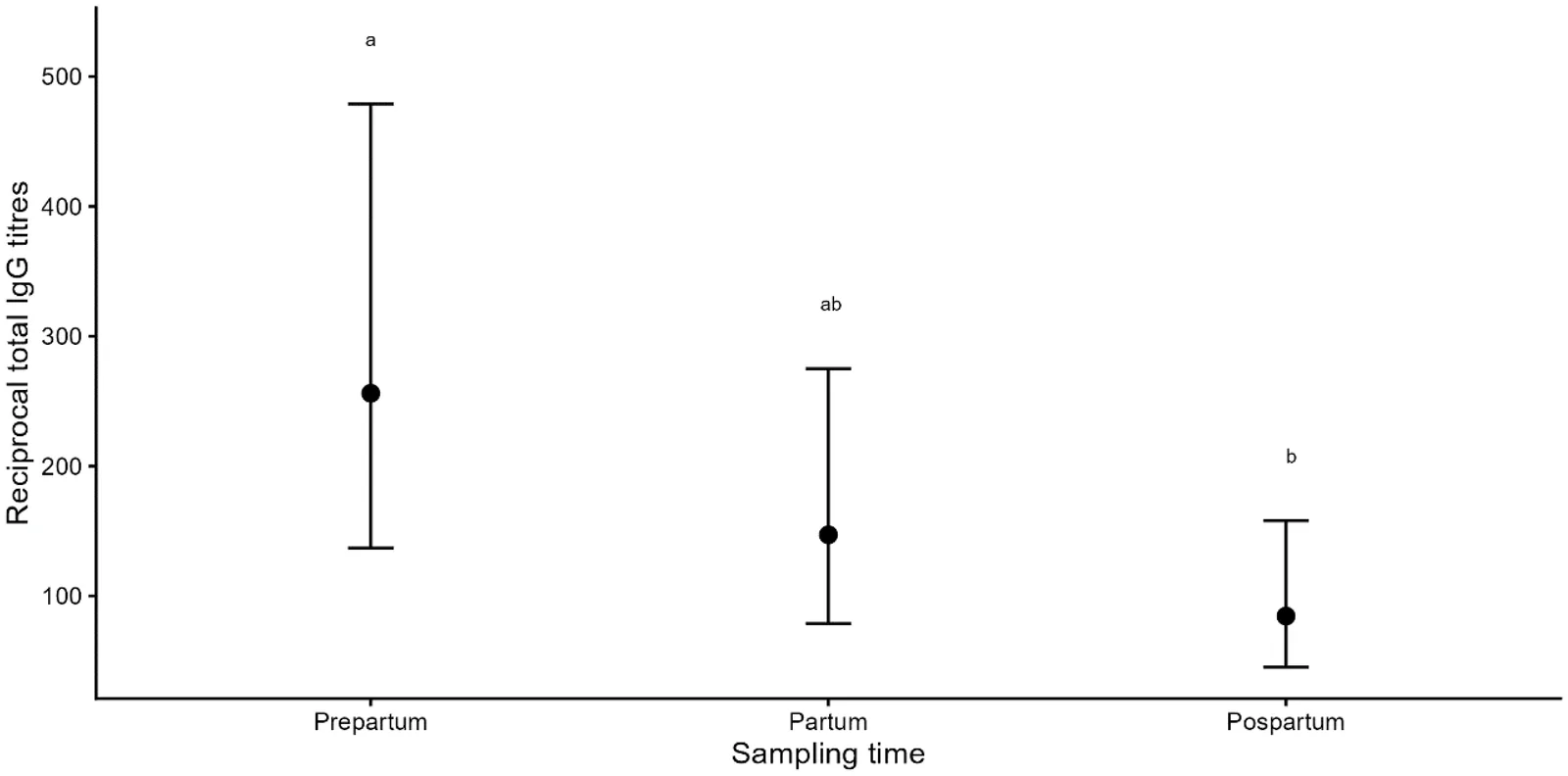
Temporal dynamics of total IgG concentrations in *N. caninum*-seropositive heifers. Total serum IgG levels showed a progressive decline from prepartum to postpartum. Statistical analysis revealed significant differences between prepartum and postpartum sampling times (P<0.05), while the concentration at parturition represented an intermediate state. This downward trend is associated with the physiological and immunological shifts characteristic of the periparturient window in dairy cattle. Data are presented as mean ± SD. Different lowercase letters indicate significant pairwise differences.

Total anti-*N. caninum* IgG reciprocal titers showed a progressive decline over the sampling period (p < 0.01; **Figure 1**). Back-transformed estimated mean reciprocal titer decreased from 256 ± 72.4 during prepartum to 147 ± 41.6 at parturition and 84.4 ± 23.9 at postpartum.

### 3.3 IgG_1_/IgG_2_ ratio

**Figure 2:**
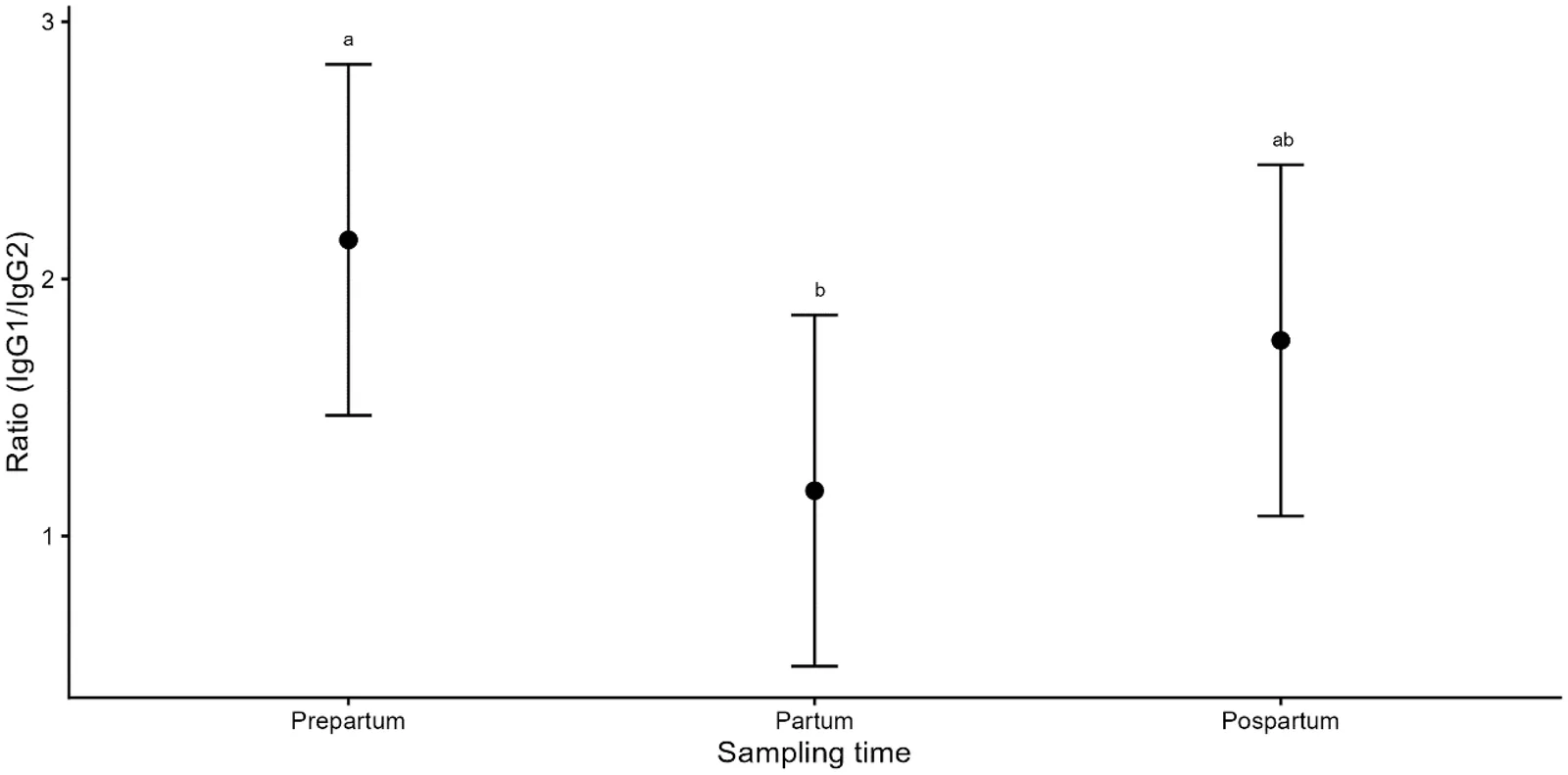
Fluctuations in the serum IgG1/IgG2 ratio against *Neospora caninum* during the transition period. The ratio of specific antibody subclasses was evaluated in congenitally infected primiparous heifers (n = 6). A significant reduction in the IgG1/IgG2 ratio was observed at the time of parturition compared to the prepartum period (P<0.05), followed by a partial recovery in the postpartum phase. This transient drop reflects the selective systemic depletion of IgG1 due to its massive transfer into the mammary gland during colostrogenesis. Data are expressed as mean ± SD. Different lowercase letters indicate statistically significant differences (P<0.05).

Statistical differences in the IgG_1_/IgG_2_ ratio were detected across the three sampling times in seropositive pregnant heifers (p < 0.001; **Figure 2**). The ratio was highest at prepartum (2.15 ± 0.305), decreased significantly at calving 1.18 ± 0.305), and showed a partial but non-significant recovery at postpartum (1.76 ± 0.305).

### 3.4 Avidity

**Figure 3:**
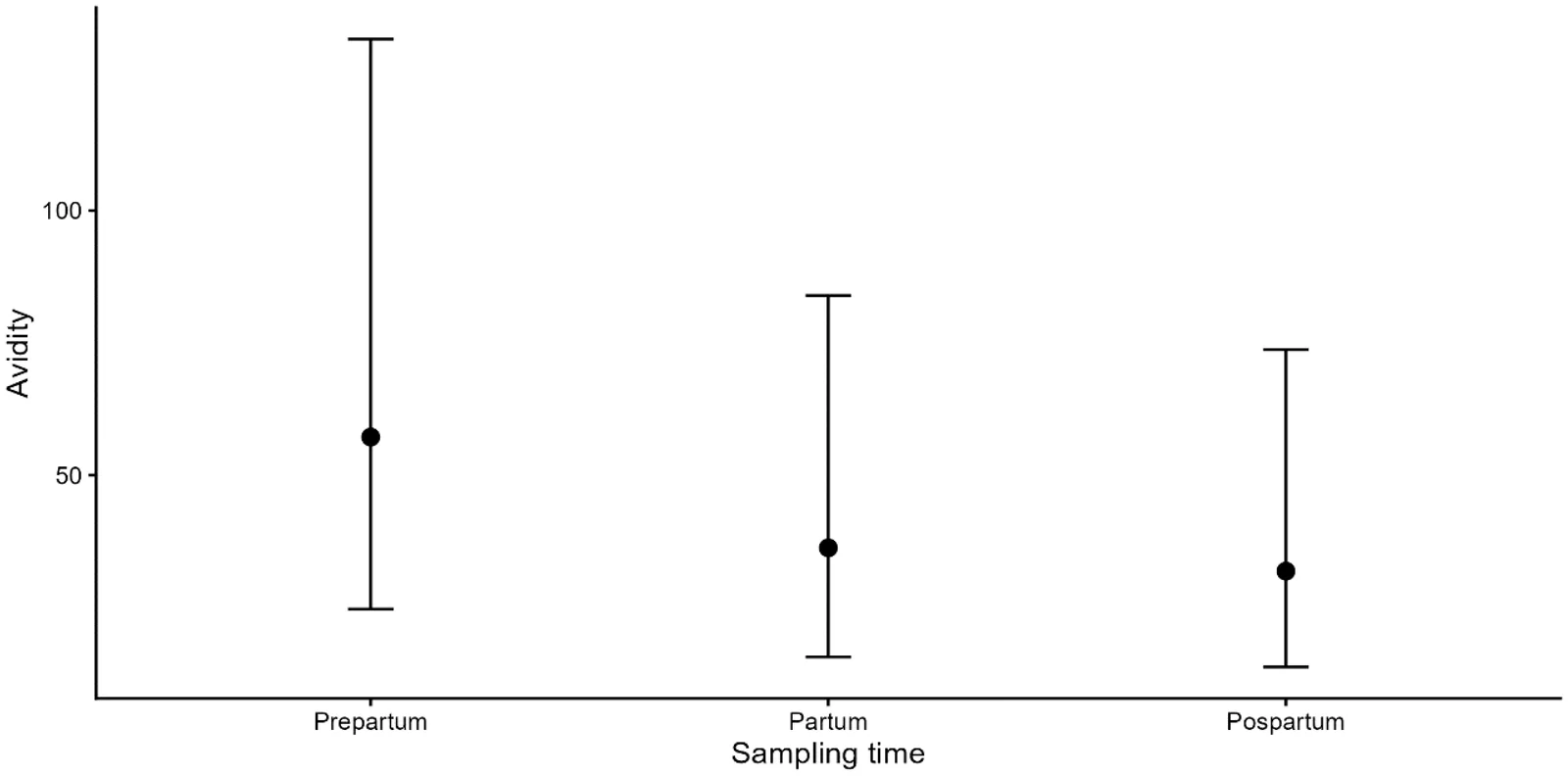
Stability of anti-*Neospora caninum* IgG avidity across the periparturient period. Specific antibody avidity was measured to assess the maturation of the humoral response. Although a slight decreasing trend was observed from prepartum to postpartum, no statistically significant differences were detected between the sampling time points (P>0.05). The maintenance of high-to-intermediate avidity levels confirms the presence of a robust and stable immune memory in chronically infected primiparous animals. Data are expressed as mean ± SD.

No differences in avidity were detected from prepartum to postpartum (**Figure 3**; p = 0.32). Estimated mean avidity values were 57.2 ± 21.3% during prepartum, 36.3 ± 13.5% at parturition, and 31.9 ± 11.8% at postpartum, indicating substantial inter-animal variability. Although mean avidity tended to decline over time, the observed values remained highly heterogeneous among individuals.

### 3.5 *Neospora caninum* evaluation in blood samples

None of the blood samples tested positive to *N. caninum* by nPCR.

### 3.6 Metabolites

**Figure 4:**
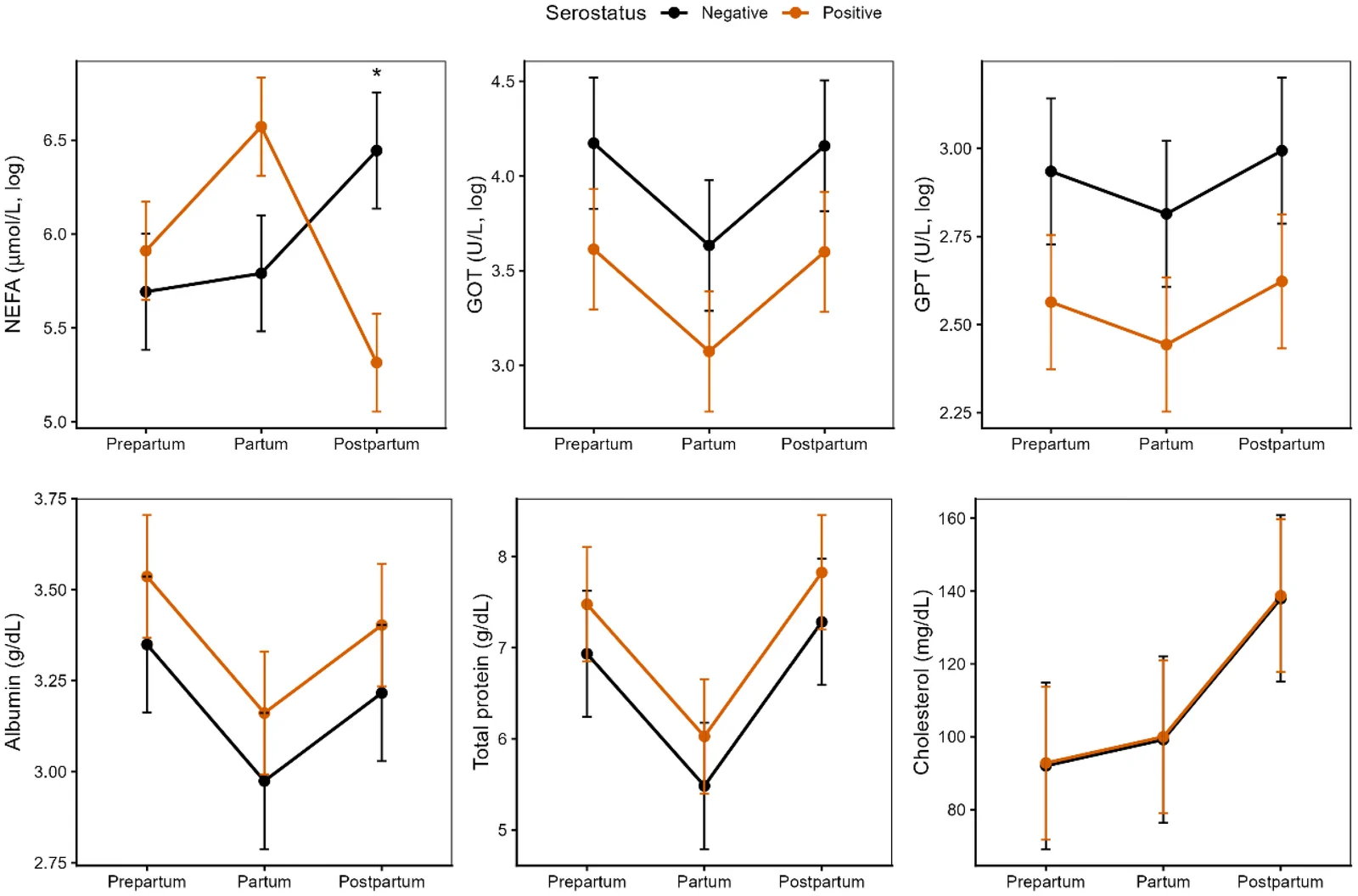
Changes in metabolic profile of dairy heifers seropositive or seronegative for *Neospora caninum* during the transition period. Data are presented as mean ± SE.

The metabolic profile of heifers varied across the transition period, whereas differences between serostatus groups were generally limited (**Figure 4**). The interaction between serostatus and time was evident in the pattern of NEFA concentrations across the evaluated periods (p = 0.03). Although numerical differences between groups were observed at parturition, these were not statistically significant, likely due to the variability within the groups. However, the temporal trajectories differed between serostatus, the seropositive group showed an increase in NEFA concentrations reaching a peak at parturition followed by a marked decline postpartum, whereas the seronegative group exhibited a more gradual increase over time, with the highest values observed postpartum. This divergence resulted in a significant statistical difference between seropositive and seronegative heifers in the postpartum period (**Figure 4**).

For GOT and GPT, no interaction between serostatus and time was detected (p > 0.05). GOT concentrations were not affected by serostatus (p = 0.10) or sampling time (p = 0.32). In contrast, GPT showed a tendency toward lower values in seropositive cows (p = 0.07), with no effect of sampling time (p = 0.75). Albumin and total protein concentrations were not influenced by serostatus (p = 0.35 and p = 0.46, respectively). Sampling time did not affect albumin concentrations (p = 0.15), whereas total protein was affected by time, decreasing at parturition compared to late gestation and partially recovering postpartum (p = 0.045). Cholesterol concentrations were not influenced by either serostatus or sampling time (p = 0.97 and p = 0.19, respectively).

## 4 Discussion

The progressive decline in total anti-*N. caninum* IgG titers from prepartum to postpartum observed in this study is consistent with previous evidence of a dynamic humoral response in naturally infected cattle (14), and extends earlier findings restricted to experimentally infected heifers and their calves (5). While that study characterized antibody transfer through colostrum and IgG_1_/IgG_2_ patterns in calves, the present results show for the first-time a within-dam temporal profile across prepartum, calving, and postpartum sampling points in naturally infected animals.

The significant reduction in IgG_1_/IgG_2_ ratio at calving, with partial recovery postpartum, indicates that the peripartum period is associated with a transient shift in antibody subclass balance rather than a stable humoral state. This shift must be interpreted within the context of gestational recrudescence, where latent bradyzoites can reactivate to tachyzoites during late pregnancy (15, 16). A Th1-type response, characterized by bovine IgG2 production, is crucial for controlling parasitemia. The transient reduction in the IgG_1_/IgG_2_ ratio around calving suggests a relative maintenance of protective IgG_2_ levels despite periparturient immunosuppression. However, because subisotype levels were determined using a single-dilution (1:10) indirect ELISA rather than full endpoint titrations due to sample volume constraints, these ratios should be interpreted with caution. This finding aligns with the general framework describing the transition period as a stage of altered immunocompetence in dairy cattle (8), although the present data do not allow identification of the specific factors driving this shift, since no immune effector or endocrine parameter linked to this change was measured.

The lack of significant differences in avidity across sampling times, despite high inter-animal variability, suggests that this parameter behaves independently from total IgG titer and subclass ratio dynamics during the transition period. IgG avidity testing is a useful tool to differentiate between recent and chronic *Neospora caninum* infections, as avidity increases progressively over time following antigen exposure, reflecting the maturation of the humoral immune response. Low-avidity antibodies are typically associated with recent or acute infection, whereas high-avidity antibodies indicate that sufficient time has elapsed for affinity maturation to occur, and are therefore considered indicative of a chronic or long-standing infection. In the present study, 4 out of 5 seropositive heifers exhibited an IgG avidity index above 50%, supporting the interpretation that these animals were undergoing a chronic stage of infection rather than a recent exposure. (13, 17). This classification is further supported by the heifers’ stable, long-term seropositive history recorded during twice-yearly pre-breeding screenings and the consistently negative nested PCR results, which point to a controlled, latent infection rather than active systemic parasitemia (18). This finding had not been previously described in naturally infected cattle and represents a novel observation of this study.

On the other hand, the decrease in the IgG_1_/IgG_2_ ratio observed around parturition in the naturally infected heifers of the present study could be also be partially explained by the selective transfer of IgG_1_ from the systemic circulation into the mammary gland during colostrogenesis, a process that is known to markedly reduce serum IgG_1_ concentrations in the peripartum period. This finding is consistent with previous work by our group in beef heifers experimentally challenged with *N. caninum*, in which infected animals also displayed IgG_1_/IgG_2_ ratios <1 together with high specific IgG avidity, while their colostrum showed high IgG_1_ concentrations and elevated avidity values, confirming the preferential enrichment of high-avidity IgG_1_ in colostrum. Furthermore, in that same study, calves born to infected heifers initially exhibited IgG_1_/IgG_2_ ratios >1 and lower avidity values before colostrum intake, which shifted toward higher IgG_1_/IgG_2_ ratios and increased avidity following colostrum ingestion, further supporting the selective transfer of high-avidity IgG_1_ from the dam to the offspring through colostrum. Taken together, these findings reinforce the notion that the decline in serum IgG_1_/IgG_2_ ratio observed around calving in naturally infected heifers reflects the physiological redirection of high-avidity IgG_1_ antibodies toward the mammary gland as part of the colostrogenesis process, rather than a change in the overall immune status of the dam.

Regarding metabolic indicators, the absence of a serostatus per time interaction for GOT, GPT, albumin, total protein, and cholesterol indicates that chronic *N. caninum* infection was not associated with altered hepatic or nitrogen metabolism markers during the transition period, in line with the general metabolic and hepatic function indicators proposed for this period (9, 19). The significant serostatus × time interaction detected for NEFA concentrations, with divergent temporal trajectories between seropositive and seronegative heifers, was the only metabolic parameter showing a serostatus-associated pattern, occurring against the background of physiological negative energy balance and fat mobilization characteristic of the transition period (6, 7). Although the leave-one-out sensitivity analysis confirmed that this interaction was not driven by a single animal, this finding should be interpreted with caution and requires further confirmation. First, the limited number of animals per group (n = 7 seropositive, n = 5 seronegative) constrains the statistical power to detect a robust and generalizable effect. Second, NEFA concentrations are influenced by multiple physiological and management-related variables that were not accounted for in this study, which limits the extent to which this interaction can be attributed specifically to serostatus.

Overall, the humoral immune parameters (total IgG decline, IgG_1_/IgG_2_ shift at calving), and the metabolic profile evaluated (limited association with serostatus, except NEFA) appear to represent largely independent processes during the transition period in chronically *N. caninum*-infected heifers.

Although the present study characterized changes in humoral immune parameters during the transition period, cell-mediated immune responses were not evaluated. Previous work has shown that DTH skin testing can detect CMIR in *N. caninum*-exposed cattle even when specific antibodies remain below detectable levels (20), indicating that humoral and cellular responses do not necessarily co-occur and that DTH may identify infected animals otherwise missed by serological testing. Given that the transition period is associated with immunosuppression and altered immunocompetence, whether CMIR, and specifically DTH responsiveness, is similarly modulated during this critical window in chronically infected heifers remains unknown. This represents a relevant gap, since the humoral changes described here cannot be assumed to reflect the behavior of cellular immunity, and future studies including DTH skin testing during the transition period are needed to determine whether cell-mediated responses follow a pattern independent of, or coupled to, the humoral dynamics observed in this study. Furthermore, because calf infection status was not determined in this trial, future investigations are required to evaluate how these maternal transition-period immune fluctuations correlate with vertical transmission rates.

## 5 Conclusions

Chronically *N. caninum*-infected Holstein heifers show a dynamic humoral response during the transition period, characterized by a progressive decline in total specific IgG and a transient reduction in the IgG_1_/IgG_2_ ratio at calving, while antibody avidity remains unaffected. Metabolic indicators of hepatic and nitrogen metabolism were not associated with serostatus, and the serostatus-associated pattern observed for NEFA requires confirmation given the limited sample size and the multiple factors known to influence this parameter. These findings indicate that the humoral immune response and the metabolic profile evaluated behave largely as independent processes during the transition period in this population.

## Data Availability

The raw data supporting the conclusions of this article will be made available by the authors, without undue reservation.
